# Identification of MUC5B as a lymph node metastasis-associated gene in lung adenocarcinoma through integrated transcriptomic and machine learning approaches

**DOI:** 10.3389/fimmu.2025.1666240

**Published:** 2025-12-02

**Authors:** Weijian Song, Qian Yang, Minjun Du, Jiacong Wei, Boxuan Zhou, Jianwei Shi, Linchuan Liang, Zixu Liu, Mei Liang, Mianyang Li, Yushun Gao

**Affiliations:** 1 Department of Thoracic Surgery, China-Japan Friendship Hospital, Beijing, China; 2 National Center for Respiratory Medicine, Beijing, China; 3 Medical School of Chinese PLA, Beijing, China; 4 Department of Thoracic Surgery, National Cancer Center/National Clinical Research Center for Cancer/Cancer Hospital, Chinese Academy of Medical Sciences and Peking Union Medical College, Beijing, China; 5 Department of Pathology, National Cancer Center/National Clinical Research Center for Cancer/Cancer Hospital, Chinese Academy of Medical Sciences and Peking Union Medical College, Beijing, China; 6 Department of Laboratory Medicine, The First Medical Center of Chinese PLA General Hospital, Beijing, China

**Keywords:** MUC5B, lung adenocarcinoma, lymph node metastasis, prognosis, machine learning

## Abstract

**Background:**

Lung adenocarcinoma (LUAD) is the most prevalent subtype of lung cancer, with lymph node metastasis serving as a key prognostic factor. MUC5B, a member of the mucin family, has been implicated in the progression of various cancers, yet its specific role in LUAD metastasis remains underexplored. This study aimed to investigate the role of MUC5B in LUAD progression and its potential as a biomarker for lymph node metastasis.

**Methods:**

We integrated TCGA data, single-cell RNA-seq, and machine learning (LASSO, SVM-RFE) to identify MUC5B and associated metastatic markers. A 13-gene predictive model was constructed and validated using ROC analysis. Immunohistochemical staining confirmed the expression of MUC5B in the clinical case samples (n=65). *In vitro* experiments were performed using MUC5B-knockdown LUAD cell lines (A549, H1975) to assess changes in proliferation, migration, invasion, and colony formation. RNA sequencing was conducted to explore downstream molecular changes following MUC5B depletion.

**Results:**

MUC5B was significantly upregulated in LUAD with lymph node metastasis and associated with poor overall and progression-free survival. Knockdown of MUC5B suppressed LUAD cell proliferation, migration, and invasion. The 13-gene model showed high predictive accuracy (AUC > 0.9) for lymph node metastasis. GSVA analysis revealed most model genes correlated positively with Th2 cells and negatively with mast cells, type II interferons. Transcriptomic profiling revealed that MUC5B depletion led to significant downregulation of GINS1, GINS2, and GINS4—core components of the DNA replication GINS complex—suggesting a regulatory axis between MUC5B and cell cycle progression. Enrichment analyses further indicated that MUC5B promotes LUAD metastasis via pathways involved in DNA replication, cell cycle, and metabolic reprogramming.

**Conclusion:**

MUC5B facilitates LUAD lymph node metastasis, potentially by regulating the GINS complex and promoting oncogenic signaling. These findings highlight MUC5B as a promising biomarker and therapeutic target for advanced LUAD.

## 1 Background

Lung cancer is the leading cause of cancer-related mortality globally, with adenocarcinoma as the most common subtype (1). In 2024, approximately 234,580 new cases and 125,070 deaths are projected in the United States (2). Lymph node metastasis is a key prognostic factor in lung cancer. The presence of lymph node involvement signifies advanced disease (stage II or higher) and is associated with poorer survival outcomes (3). For instance, the five-year survival rate for stage I lung cancer without nodal involvement ranges from 70-90%, but this drops significantly with lymph node metastasis. Patients with N1, N2, and N3 disease show five-year survival rates of 40-60%, 20-30%, and less than 10%, respectively (4). Current diagnostic tools, such as computed tomography (CT), positron emission tomography (PET), and endobronchial ultrasound-guided transbronchial needle aspiration (EBUS-TBNA), though useful, remain insufficient in fully predicting metastasis (5). Consequently, identifying novel biomarkers and molecular targets is crucial for improving prognosis.

The MUC family consists of large, highly glycosylated proteins produced by epithelial cells, characterized by tandemly repeated amino acid sequences. These glycoproteins, encoded by the mucin gene family, play crucial roles in protecting and lubricating epithelial surfaces across various systems, including the respiratory, digestive, and reproductive systems (6). Mucins can be classified as secreted (e.g., MUC5AC, MUC5B) or membrane-bound (e.g., MUC1, MUC4, MUC16) (7). Secreted mucins form mucus layers that trap pathogens, while membrane-bound mucins act as protective barriers. Dysregulation in mucin expression is associated with chronic inflammatory conditions and cancers (8).

The MUC5B gene, located at cytogenetic position 11p15.5, encodes a large secreted mucin with a molecular mass of 596,340 Da (9). Under normal conditions, MUC5B helps maintain the integrity of the mucosal barrier. It has gained attention in benign lung diseases such as idiopathic pulmonary fibrosis (IPF), chronic obstructive pulmonary disease (COPD), and asthma, where excessive mucus production obstructs airways and contributes to inflammation (10, 11).

In LUAD, MUC5B expression is associated with poor differentiation, advanced TNM stages, and worse prognosis, confirmed by multivariable analyses showing a higher risk of death (12–14). MUC5B is positively correlated with cancer-associated fibroblasts and myeloid-derived suppressor cells in the tumor microenvironment (TME), indicating a role in TME-mediated tumor progression (15). In lung invasive mucinous adenocarcinoma (IMA), MUC5B expression is higher compared to non-mucinous adenocarcinoma. This is regulated by transcription factors such as FOXA3, SPDEF, and HNF4α, which promote mucin expression in mucinous lung cancer cells, especially in cases with KRAS mutations (16). Additionally, MUC5B-AS1, a lncRNA upregulated in LUAD, enhances cancer cell migration and invasion by forming an RNA-RNA duplex with MUC5B, further promoting its expression (17).

Machine learning techniques, including weighted gene co-expression network analysis (WGCNA), least absolute shrinkage and selection operator (LASSO) and support vector machine-recursive feature elimination (SVM-RFE), were applied to uncovering molecular signatures involved in prognostic predictive factors and refining diagnostic models in a wide range of malignant diseases (18–20). These methods assist in selecting relevant diagnostic variables by narrowing down high-dimensional data.

Bioinformatics and machine learning strategies are increasingly recognized for their application in lung cancer research. These methods have been pivotal in elucidating the roles of EGFR mutations in LUAD, which are known to influence drug resistance in targeted therapies (21). Similarly, STK11 mutations have been associated with responses to immune therapies in LUAD, highlighting the critical interplay between genetic mutations and the tumor microenvironment (TME) (22). Notably, machine learning algorithms have also been used to uncover key features of the TME that contribute to anti-tumor immunity, demonstrating the importance of integrating computational tools with clinical data.

In this study, we utilized an integrated bioinformatics approach and machine learning strategies to analyze LUAD single-cell RNA sequencing data and gene expression datasets obtained from the GEO and TCGA databases. The aim was to identify and screen key biomarker genes associated with lymph node metastasis in LUAD. Additionally, we performed *in vitro* validation experiments to support the findings. These results provide prospective targets for the diagnosis, prevention, and prognostic assessment of lymph node metastasis in LUAD.

## 2 Materials and methods

### 2.1 Data source and processing

Single-cell RNA-seq data (GSE127465) from GEO database (7 LUAD samples, 31,179 cells) were analyzed alongside TCGA data (515 tumors, 59 normals). External validation employed GEO datasets GSE13213 and GSE11969.

### 2.2 Single-cell data processing

Single-cell data were processed using MAESTRO v1.5.1, including quality control, batch correction, clustering, and annotation (23). Cells with <1,000 reads or <500 genes were excluded. PCA (top 2,000 genes) and clustering (KNN/Louvain; 30 PCs, resolution=1) were performed, followed by t-SNE visualization (25 clusters). DEGs were identified (Wilcoxon; |logFC|≥0.25, FDR<1×10^-5^) and annotated using published markers.

### 2.3 Identification of differentially expressed genes in lymph node metastasis

TCGA LUAD samples were stratified into lymph node-positive (N1+N2+N3) and -negative (N0) groups. DEGs were analyzed using “limma”, with comparative assessment of MUC5B versus other MUC family genes. Survival analysis was performed after dichotomizing patients by median MUC5B expression.

### 2.4 Weighted gene co-expression network analysis

We performed WGCNA on lung adenocarcinoma samples to identify modules of genes associated with lymph node metastasis (24). The expression matrix was filtered using “goodSamplesGenes” (soft threshold=10), identifying 12 gene modules. Genes with module membership>0.7 and gene significance>0.1 were retained for lymph node metastasis analysis. Pearson correlation identified candidate genes associated with both MUC5B and metastasis.

### 2.5 Protein-protein interaction network analysis

A PPI network was constructed using GeneMANIA (25), with hub genes identified (degree>30) and further screened by MNC/Betweenness centrality. NetworkAnalyst analyzed miRNA-MUC5B (TarBase v8.0) and TF-MUC5B (JASPAR) interactions, integrated into a miRNA-MUC5B-TF network via Cytoscape. Functional enrichment was performed using GO/KEGG.

### 2.6 Machine learning algorithms

Core genes were analyzed using LASSO (“glmnet”) and SVM-RFE (“e1071”) (20) for feature selection. SHAP visualized model interpretations, while “pROC” calculated AUC values. Nomograms (“rms”) and decision curves (“rmda”) were generated for clinical utility assessment.

### 2.7 Relationship between core genes and immune cells

Immune-related pathways were identified through literature review. “GSVA” calculated immune scores, with significant changes assessed (Pearson’s test, P<0.05). Correlations between immune scores/diagnostic genes and core genes/immune infiltration were analyzed. For immune pathway correlation analyses, statistical significance was determined using the p.adjust function in R to correct for multiple comparisons (26).

### 2.8 Human LUAD samples

This retrospective study analyzed 65 LUAD patients diagnosed at the Cancer Hospital, Chinese Academy of Medical Sciences (January 2017-December 2018). Inclusion criteria: 1) surgical treatment; 2) pathological LUAD confirmation; 3) complete clinical/follow-up data. Exclusion criteria: 1) other malignancies; 2) non-LUAD pathology; 3) lost to follow-up. The study was approved by the Institutional Ethics Committee (NCC2019C-167). All patients underwent 5-year postoperative follow-up (or until death) with informed consent.

### 2.9 Immunohistochemistry and analysis

For IHC analysis, LUAD specimens were deparaffinized in xylene and rehydrated through graded alcohols. After endogenous peroxidase blocking (3% H_2_O_2_) and antigen retrieval (citrate buffer), sections were incubated with anti-MUC5B antibody (1:100, HUABIO) at 4°C overnight, followed by HRP-conjugated secondary antibody (30 min, RT). DAB (Servicebio) was used for visualization. Staining was quantified using the H-score formula: H-Score = Σ (pi × i) = (% weak cells × 1) + (% moderate cells × 2) + (% strong cells × 3), where i represents staining intensity (0=negative, 1=weak, 2=moderate, 3=strong) and pi is the percentage of cells at each intensity level (27, 28). The IHC scoring was independently performed by two senior pathologists with extensive experience in pathological diagnosis of thoracic tumors.

### 2.10 Cell culture and siRNA transfection

H1975 and A549 cells were maintained in RPMI-1640 and DMEM (10% FBS) at 37°C/5% CO_2_, respectively. Cells were transfected with either negative control (NC) or MUC5B-targeting siRNA (siMUC5B; GenePharma) using the following sequences: Sense: 5′-GCAGCUACGUUCUGUCCAATT-3′; Antisense: 5′-UUGGACAGAACGUAGCUGCTT-3′. Transfected cells were cultured under standard conditions until analysis.

### 2.11 RNA extraction and qRT-PCR

Total RNA was extracted from tissues or cells with TRIzol (Invitrogen, USA), according to the manufacturer’s instructions. Total RNA was reverse transcribed using a reverse transcription kit (Vazyme Biotech Co., Ltd, China), according to the manufacturer’s protocol. β-actin was used to normalize the RNA levels using the 2-ΔΔCt method. The sequences of the primers for qRT-PCR were: MUC5B-F: 5′- CCCGTGTTGTCATCAAGGC -3′; MUC5B-R: 5′- CAGGTCTGGTTGGCGTATTTG -3′; β-actin-F: 5′- TGGCACCCAGCACAATGAA-3′; β-actin-R: 5′- CTAAGTCATAGTCCGCCTAGAAGCA-3′; GINS1-F: 5′-ACGAGGATGGACTCAGACAAG-3′; GINS1-R: 5′-TGCAGCGTCGATTTCTTAACA-3′; GINS2-F: 5′-CCCTGGTTTACCCGTGGAAG-3′; GINS2-R: 5′-GGGAGCAGGCGACATTTCT-3′; GINS3-F: 5′-ACTTTTATCGGACGTTTTCGCC-3′; GINS3-R: 5′-TCTCCATCTCGTCTAGCCTGG-3′; GINS4-F: 5′-AGTTGGCCTTTGCCAGAGAG-3′; GINS4-R: 5′-GAACTGCCCGAAAGAGGTCC-3′.

### 2.12 CCK-8 assay

Cell growth was measured using a CCK-8 kit (Dojindo, Kumamoto, Japan) according to the instructions. Briefly, approximately 1×10^3^ cells per well were plated into 96-well plates and cultured in the indicated medium. Cell proliferation, measured at 450 nm, was examined every day for four days according to the manufacturer’s protocol according to the manufacturer’s protocol.

### 2.13 Colony formation assay

1×10^3^ cells per well were seeded in 6-well plates, and the culture was terminated when the cells formed obvious clones under the microscope. The colonies were fixed with 4% paraformaldehyde and stained with 0.1% crystal violet for 30 min at room temperature. Finally, the colony number was quantified.

### 2.14 Wound healing assay

H1975 and A549 cells were seeded into 6-well plates. Once the cells reached full confluence, a scratch was created using a 200 µl pipette tip. The dislodged cells were removed with serum-free medium. Images of the wounded area were captured at 0 hours and 24 hours. The cell migration rate was calculated using the following formula: %migration rate = ((0 h wound area - 24 h wound area) × 100)/0 h wound area.

### 2.15 Transwell experiment

Invasion assay:

H1975 (2.5×10^4^) and A549 (5×10^4^) cells in serum-free medium were seeded into Matrigel-coated Transwell chambers (8 µm; Corning). Lower chambers contained 10% FBS medium. After 48h incubation (37°C/5% CO_2_), non-invading cells were removed, membranes were fixed (4% PFA) and stained (0.1% crystal violet, 30min). Invading cells were counted from three random fields

Migration assay:

Performed similarly without Matrigel coating.

### 2.16 RNA-SEQ

RNA sequencing was conducted using the Illumina NovaSeq 6000 system (Shanghai Personal Biotechnology Co., Ltd). The transcriptomic data analysis workflow included several steps: initial quality filtering with *fastp* (v0.22.0) to obtain high-quality FASTQ files; read mapping to the reference genome using *HISAT2* (v2.1.0); gene expression quantification with *HTSeq* (v0.9.1), applying FPKM for normalization; identification of differentially expressed genes through *DESeq2* (criteria: fold change > 2, p < 0.05); and functional annotation using *topGO* (v2.50.0) and *ClusterProfiler* (v4.6.0) for GO and KEGG enrichment analyses. All bioinformatics analyses were performed on the GenesCloud platform provided by Personalbio (https://www.genescloud.cn).

### 2.17 Statistical analysis

Statistical analyses were performed using R (v4.2.3) and GraphPad Prism (v10.1.2). Diagnostic accuracy was evaluated via ROC curves (“survivalROC” package) with AUC calculation. Nomograms and calibration curves were generated using “rms”. Functional enrichment (GO/KEGG) was conducted with “clusterProfiler”. Continuous variables were compared using Wilcoxon rank-sum (two groups) or Kruskal-Wallis (≥3 groups) tests, while categorical variables used Fisher’s exact/Chi-square tests. Unpaired t-tests analyzed experimental comparisons. P<0.05 was considered statistically significant.

## 3 Results

### 3.1 Analysis of MUC family gene expression in different immune cells using single-cell data

**Figure 1:**
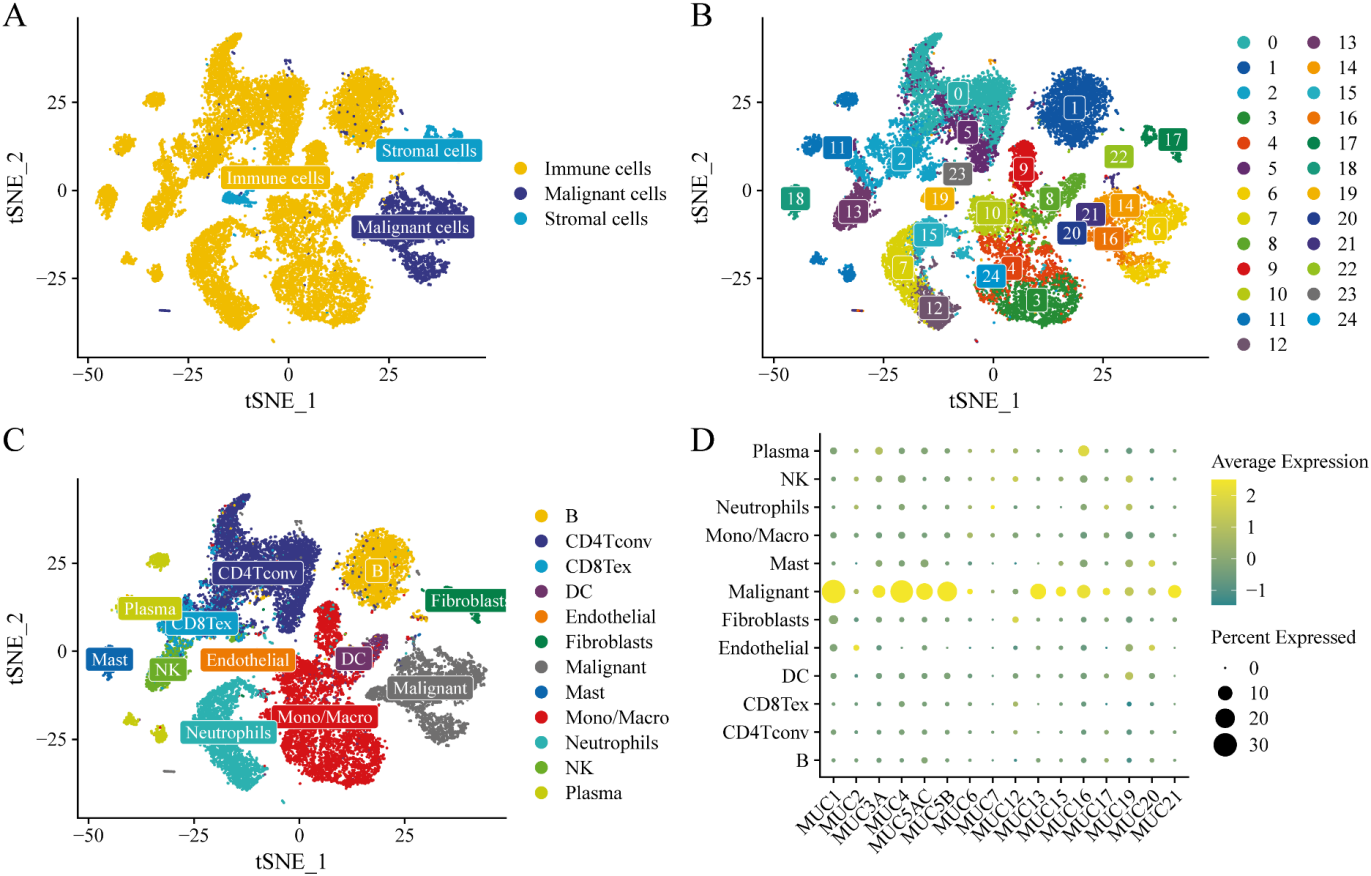
Single-cell data processing and MUC family gene expression. **(A)** t-SNE plot showing cell malignancy distribution; **(B)** t-SNE plot of 25 cell clusters; **(C)** Annotated t-SNE plot of 12 cell types; **(D)** MUC family gene expression across different cells.

After quality control, a total of 31,797 genes and 31,179 cells were obtained. We applied the MAESTRO pipeline for single-cell data analysis, identifying the top 2,000 variable features and using PCA for dimensionality reduction, KNN, and Louvain algorithms to identify clusters. To capture cell differences across varying datasets, we set the number of principal components to 30 and used a clustering resolution of 1. We employed t-SNE method to further reduce dimensions and visualize clustering results. We first determined the malignancy of these clusters, identifying 26,055 immune cells, 3,995 malignant cells, and 1,129 stromal cells, as shown in **Figure 1A**. The results of the cell clustering are displayed in **Figure 1B**, where 25 clusters were identified. The number of cells in each cluster is presented in **Supplementary Table 1**. Using the marker-based annotation method in MAESTRO, cell clusters were annotated based on differentially expressed (DE) genes, with marker genes collected from published resources. The annotated clustering results are shown in **Figure 1C**. Finally, we analyzed MUC family gene expression across different immune cells, finding the highest expression in malignant cells (**Figure 1D**). Due to dataset limitations (lacking normal/metastatic lymph node data), we analyzed peripheral blood mononuclear cells (PBMCs) and tumor cells to characterize MUC family gene expression patterns. Subgroup analyses by N-stage revealed metastatic-associated expression differences. Notably, MUC family genes showed significantly elevated expression in tumor tissues, particularly malignant cells, compared to normal cells (**Supplementary Figure 1**).

### 3.2 Identification of differentially expressed genes associated with lymph node metastasis

**Figure 2:**
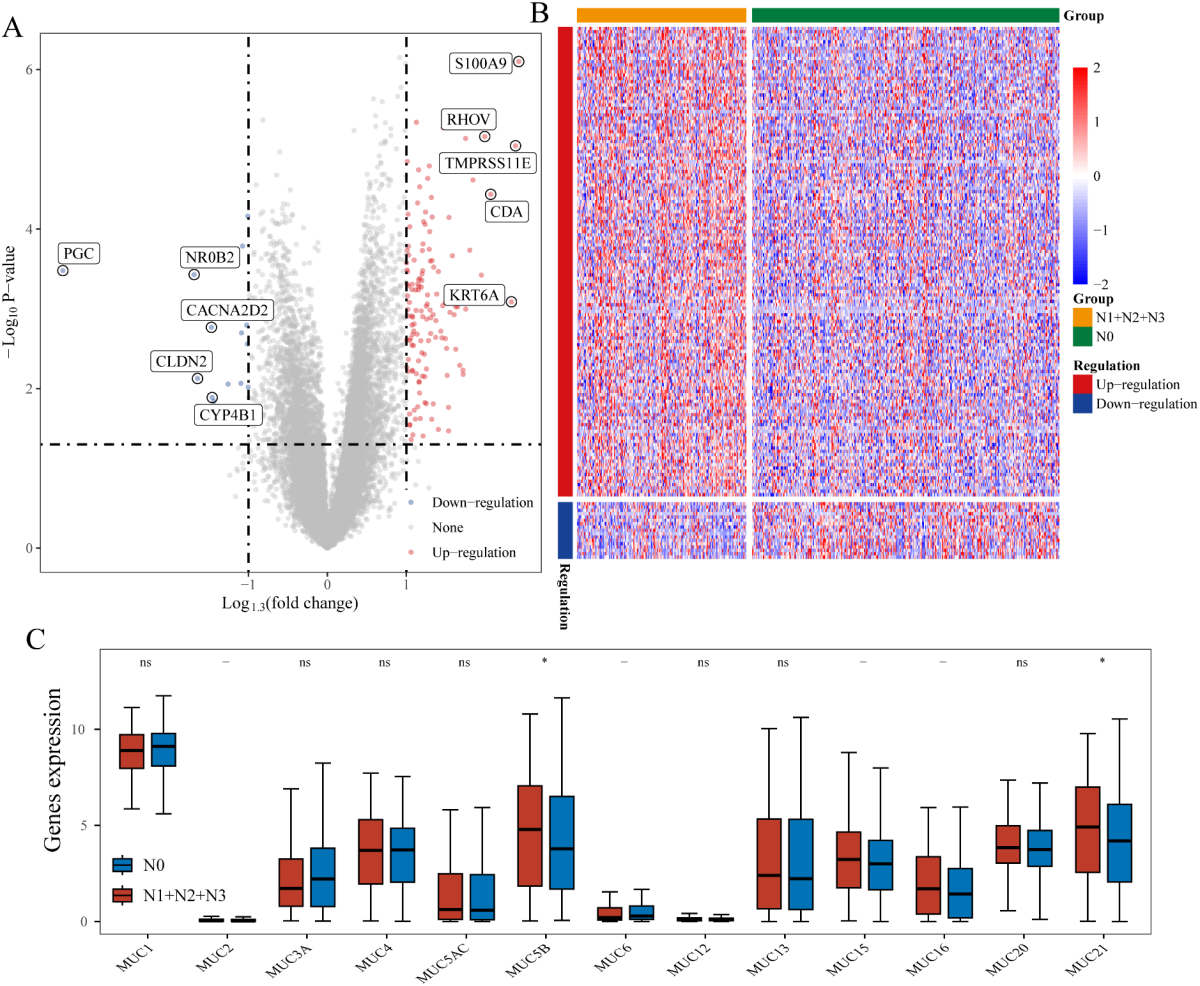
Differentially expressed genes between lymph node metastasis and non-metastasis groups. **(A)** Volcano plot of differentially expressed genes; **(B)** Heatmap of gene expression; **(C)** MUC family gene expression in metastasis and non-metastasis groups. *P < 0.05.

We performed differential gene expression analysis on lung cancer samples, identifying 158 DEGs (adj.P.Val < 0.05, |log1.3FC| > 1) between lymph node metastasis (N1+N2+N3) and non-metastasis (N0) samples. Among these, 141 genes were upregulated in the lymph node metastasis group, while 17 genes were downregulated (**Figure 2A**). A heatmap of differential gene expression is shown in **Figure 2B**. We also analyzed MUC family gene expression differences between metastasis and non-metastasis groups, finding significant differences for MUC5B and MUC21 (**Figure 2C**).

**Figure 3:**
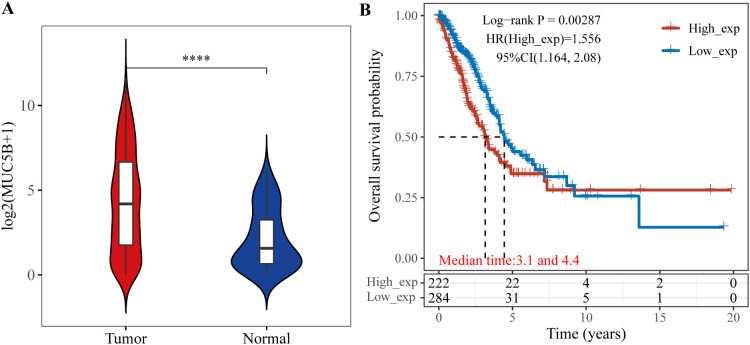
Expression of MUC5B in tumor and normal tissues and its prognostic impact on overall survival in LUAD. **(A)** MUC5B expression in tumor and normal tissues; **(B)** Kaplan-Meier survival curve for MUC5B. ****P < 0.0001.

Further analysis revealed that only MUC5B could stratify lung adenocarcinoma patients into high- and low-risk groups. The expression in tumor versus normal tissues is shown in **Figure 3A**, and the prognostic Kaplan-Meier curve is shown in **Figure 3B**. MUC21 was not significantly associated with prognosis. We extended progression-free survival (PFS) analysis using TCGA survival data, revealing significant association between MUC5B and PFS in lung adenocarcinoma (p<0.05), reinforcing its lymph node metastasis relevance (**Supplementary Figure 2**).

### 3.3 WGCNA analysis

**Figure 4:**
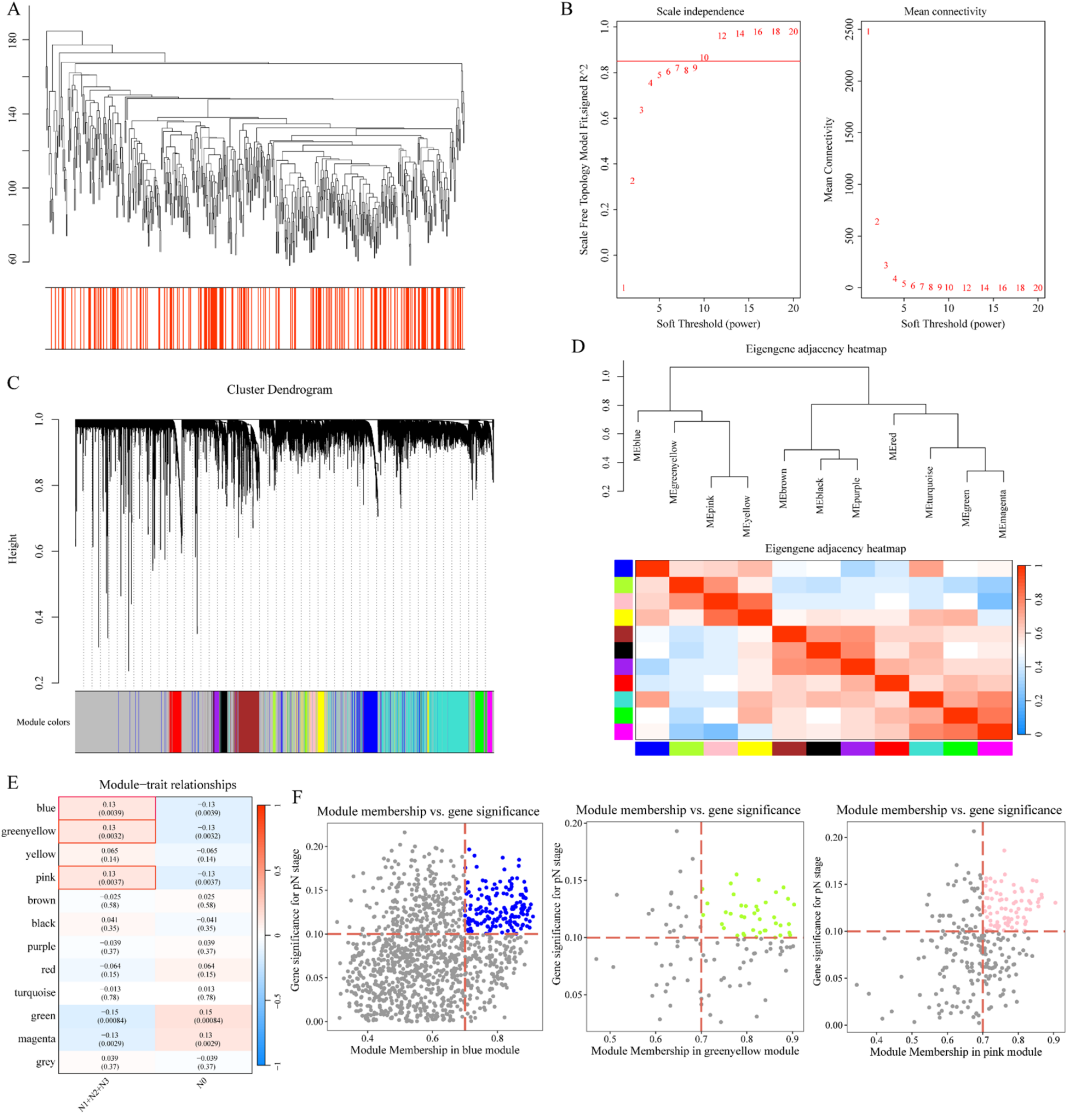
WGCNA results. **(A)** Hierarchical clustering of samples; **(B)** Network topology analysis for various soft-thresholding powers; **(C)** Gene dendrogram and module colors; **(D)** Correlation clustering of 12 modules; **(E)** Module-trait relationships; **(F)** GS vs. MM for lymph node metastasis-associated genes.

To identify genes associated with lymph node metastasis, we performed weighted gene co-expression network analysis (WGCNA) on 16,033 genes, with MAD > 0.15. Hierarchical clustering of samples is shown in **Figure 4A**. We used the R package WGCNA to construct a weighted co-expression network, with a soft threshold of 10 for module selection. The network showed characteristics of a scale-free network, with a negative correlation between the log(k) of node connectivity and the log(P(k)) of node occurrence. Using average-linkage hierarchical clustering, we grouped genes into modules, with a minimum of 50 genes per module. We identified 12 modules (**Figure 4C**), excluding the gray module (unclustered genes). Correlation clustering of these modules is shown in **Figure 4D**. We analyzed the correlation between each module and immune scores, finding strong correlations for the blue (1,217 genes), green yellow (114 genes), and pink (271 genes) modules with lymph node metastasis. Gene significance (GS) and module membership (MM) relationships are shown in **Figure 4F**. We retained genes with MM > 0.7 and GS > 0.1, identifying 276 co-expressed genes related to lymph node metastasis.

### 3.4 Identification of key genes

**Figure 5:**
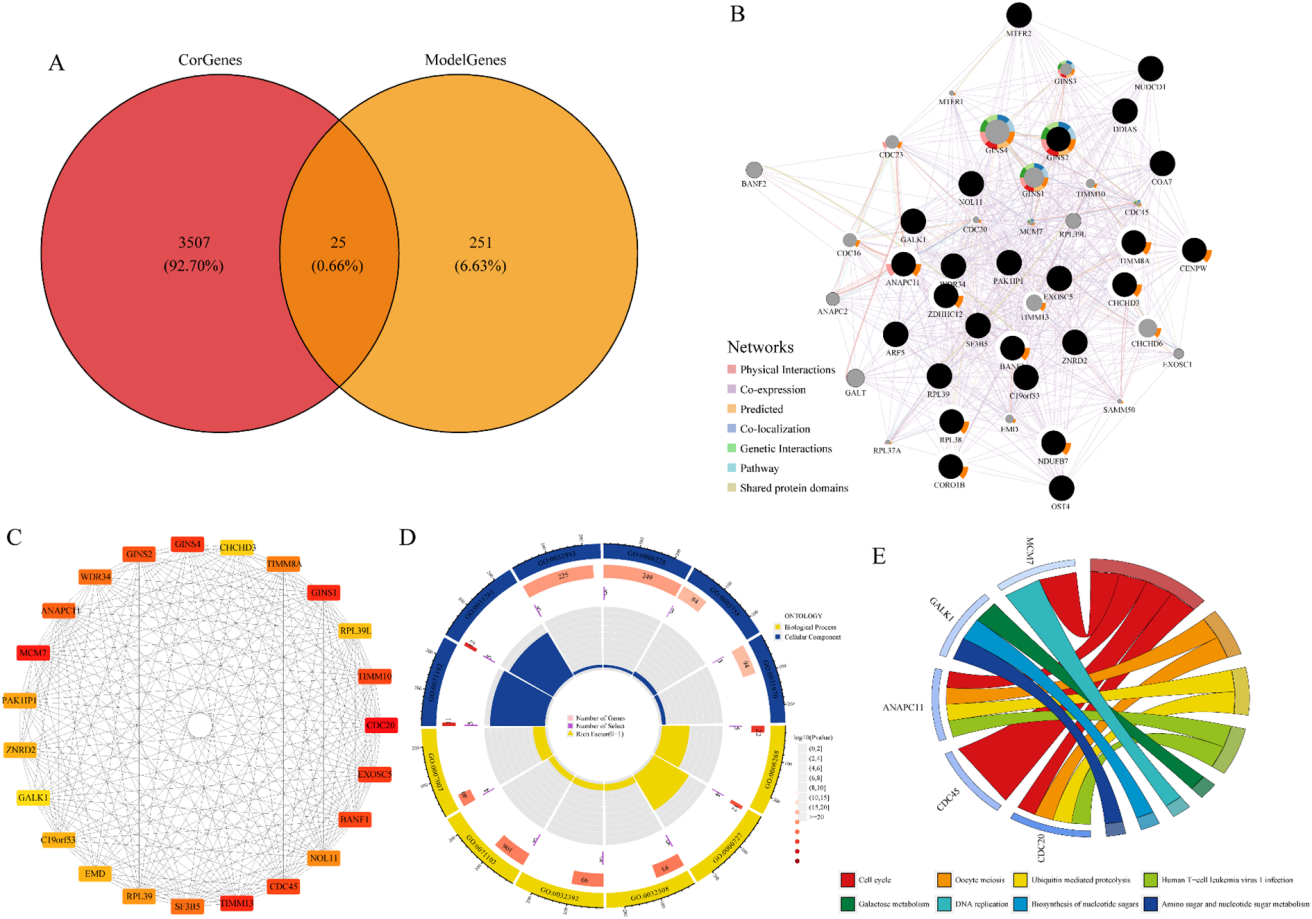
Identification of key genes and functional analysis. **(A)** Intersection of co-expressed genes related to lymph node metastasis and MUC5B; **(B)** PPI network of 25 candidate genes; **(C)** Sub-network constructed with cytoHubba; **(D)** GO functional enrichment analysis of key genes; **(E)** KEGG pathway enrichment analysis of key genes.

Pearson correlation analysis with MUC5B revealed 3,532 genes significantly associated with MUC5B, from which 25 candidate genes were identified based on overlap with lymph node metastasis-associated genes (**Figure 5A**).

Next, we used the GeneMANIA database to construct the protein-protein interaction (PPI) network of 25 candidate genes (**Figure 5B**). Using the cytoHubba plugin, we identified core genes within this network, retaining 23 genes with a degree greater than 30 as key genes (**Figure 5C**). To enhance the PPI analysis, we included Maximal Clique Centrality (MNC) and Betweenness centrality for gene screening. Key genes consistently ranked highly across all parameters (Degree, MNC, Betweenness), demonstrating their network significance (**Supplementary Figure 3**). Finally, we performed functional enrichment analysis of these key genes using GO and KEGG databases (**Figures 5D, E**). Our GO analysis reveals that the model’s key genes are significantly enriched in mitochondrial membrane organization, DNA replication/repair, and chromosomal structure maintenance pathways, suggesting two clinically relevant mechanisms underlying lung adenocarcinoma metastasis. The strong association with inner mitochondrial membrane organization and intermembrane space components indicates potential metabolic reprogramming in metastatic cells, consistent with clinical observations of enhanced oxidative phosphorylation and altered mitochondrial morphology in circulating tumor cells. The DNA-related processes, including break-induced repair and replication preinitiation, reveal molecular vulnerabilities that may explain the high mutational burden in lymph node metastases and poor chemotherapy response observed in clinical cohorts. Notably, the enrichment of protein-DNA complexes highlights potential therapeutic targets, such as PARP inhibitors for tumors with DNA repair defects and MCM complex inhibitors currently in clinical trials. These findings collectively suggest that mitochondrial dysfunction and genomic instability jointly contribute to metastatic progression, with DNA replication/repair mechanisms serving as both biomarkers for metastatic risk and potential targets for therapeutic intervention. KEGG analysis revealed key pathways including cell cycle, DNA replication, and ubiquitin-mediated proteolysis for proliferation control, along with galactose and nucleotide sugar metabolism for cellular energetics. Notably, Human T-cell leukemia virus 1 infection pathway enrichment suggests potential immune evasion mechanisms. These findings collectively indicate our model genes coordinate tumor growth through proliferation, metabolic reprogramming, and immune modulation - hallmarks of cancer progression.

### 3.5 Model construction using machine learning and relationship between key genes and immune cells

**Figure 6:**
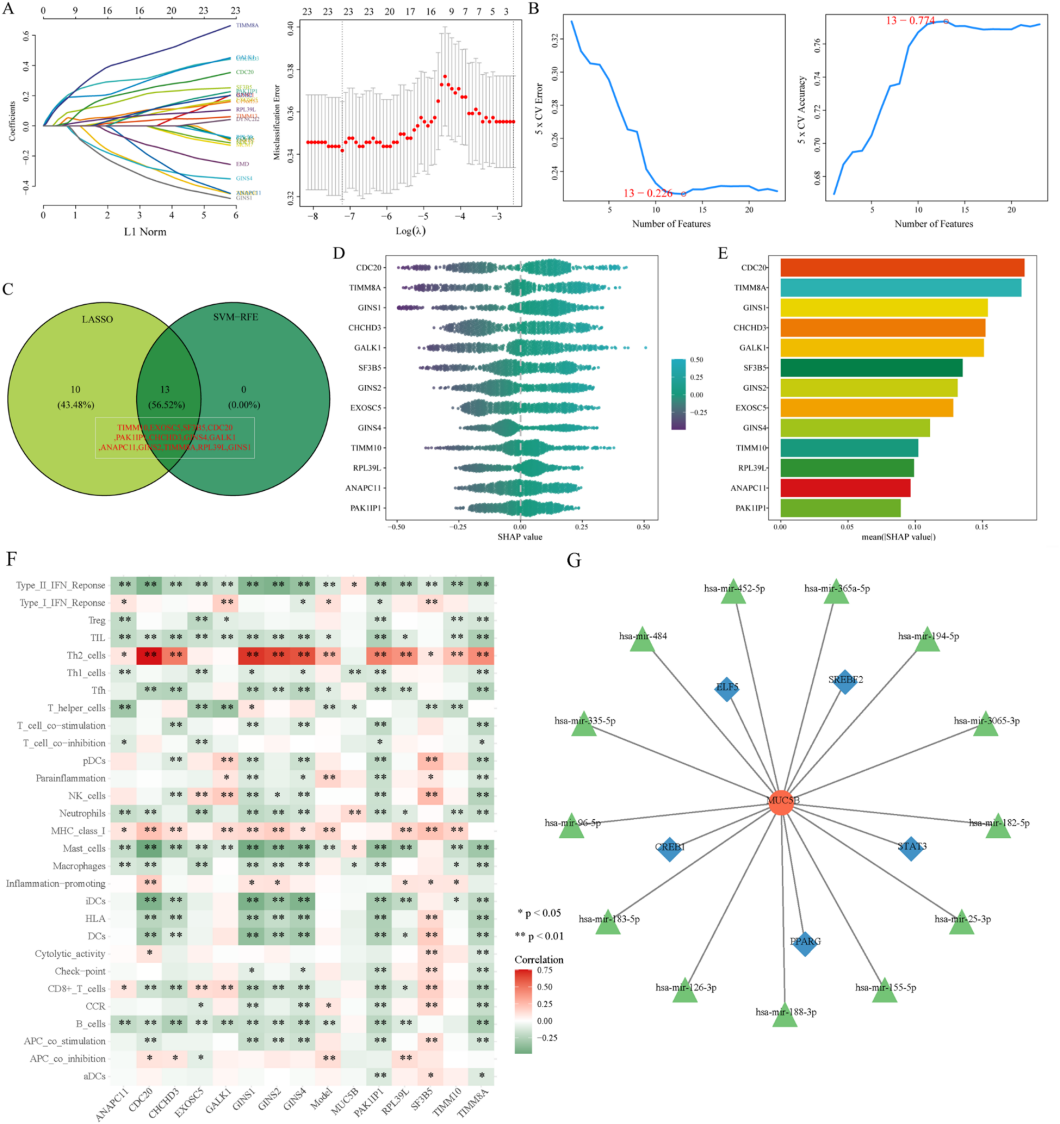
Construction of gene features and correlation between key genes and immune cells. **(A)** LASSO coefficient profile of 23 core genes; **(B)** SVM-RFE result validation using 10-fold CV RMSE; **(C)** Venn diagram showing the intersection of LASSO and SVM-selected genes; **(D)** SHAP value scores of each input feature; **(E)** SHAP summary plot showing the impact of each feature on the full model output. **(F)** Heatmap showing the correlation between the model and immune scores; **(G)** miRNA-mRNA-TF network for MUC5B.

In addition to WGCNA, two machine learning methods were used to identify predictors of lymph node metastasis in lung adenocarcinoma. LASSO regression selected features from 23 core genes, identifying 23 optimal variables (**Figure 6A**). SVM-RFE further refined the selection, yielding 13 genes (**Figure 6B**). Their intersection produced 13 key model genes (**Figure 6C**), including TIMM10, EXOSC5, SF3B5, CDC20, PAK1IP1, CHCHD3, GINS4, GALK1, ANAPC11, GINS2, TIMM8A, RPL39L, and GINS1. SHAP analysis assessed the contribution of each gene (**Figures 6D, E**).

Immune scores were calculated using GSVA, and Pearson correlation was performed between the 14 key genes (13 model genes plus MUC5B) and immune components (**Figure 6F**). Most genes showed positive correlations with Th2 cells and negative correlations with mast cells and type II interferons. Th2 cells secrete cytokines such as IL-4, IL-5, and IL-13, which not only induce the polarization of M2-type macrophages but also directly suppress the activation and proliferation of CD8^+^ cytotoxic T cells. As core anti-tumor immune cells, the impaired function of CD8^+^ T cells significantly weakens the body’s ability to kill tumor cells, thereby clearing immune obstacles for tumor metastasis. On the other hand, the negative correlation between high model gene expression and mast cells reduces the release of chemokines (e.g., CXCL10, CCL3, CCL5) by mast cells. These chemokines are key signaling molecules that recruit CD4^+^ T cells and CD8^+^ T cells to the tumor immune microenvironment; reduced expression of these chemokines leads to insufficient infiltration of anti-tumor immune cells, further weakening immune surveillance. Additionally, the negative correlation between model genes and type II interferon reduces the inhibitory effect of type II interferon on Th2 cell differentiation, while also weakening its ability to promote the maturation of antigen-presenting cells. Impaired maturation of APCs prevents effective presentation of tumor antigens to T cells, ultimately blocking the initiation of anti-tumor immune responses. These findings suggest that high expression of model genes may indicate an immunosuppressive microenvironment conducive to immune evasion.

Finally, NetworkAnalyst was used to explore miRNAs and transcription factors (TFs) regulating MUC5B (**Figure 6G**). miRNAs such as hsa-miR-484, hsa-miR-335-5p, and hsa-miR-182-5p may modulate MUC5B through interactions with TFs. These miRNAs are potentially involved in LUAD metastasis and immune evasion by affecting tumor cell adhesion and migration. Key TFs regulating MUC5B included ELF5, SREBF2, STAT3, PPARG, and CREB1, suggesting MUC5B may drive lymph node metastasis via these transcriptional networks.

### 3.6 Model evaluation and validation

**Figure 7:**
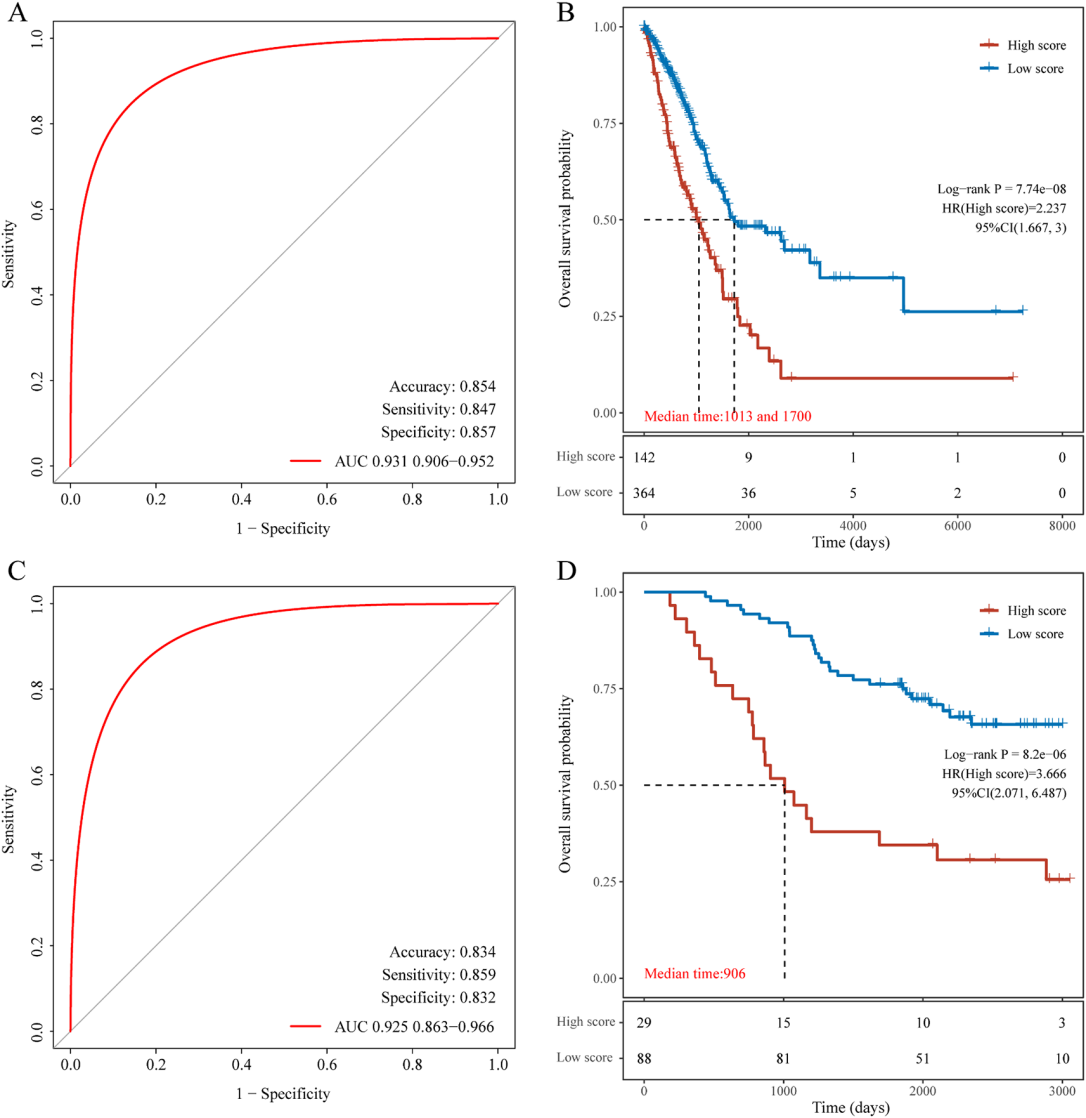
Evaluation of the diagnostic value of the 13-gene signature. **(A)** ROC curve and AUC in the training set; **(B)** Kaplan-Meier curve in the training set; **(C)** ROC curve and AUC in the validation set; **(D)** Kaplan-Meier curve in the validation set.

To further evaluate the diagnostic accuracy of the identified genes for predicting lymph node metastasis in lung adenocarcinoma, we performed ROC analysis and validated the model using the GSE13213 and GSE11969 dataset. ROC analysis demonstrated an AUC of 0.931 in the training set (**Figure 7A**). The prognostic Kaplan-Meier curve (**Figure 7B**) showed that the model successfully stratified patients into high- and low-risk groups. In the GSE13213 validation set, the model achieved an AUC of 0.925 (**Figure 7C**), and the KM curve (**Figure 7D**) confirmed the model’s ability to separate patients into distinct risk groups. AUC of ROC in GSE11969 was 0.894 (0.809–0.947). These results suggest the 13-gene model has high diagnostic and prognostic value.

### 3.7 MUC5B was specifically expressed in lymph node metastasis positive LUAD clinical samples

**Figure 8:**
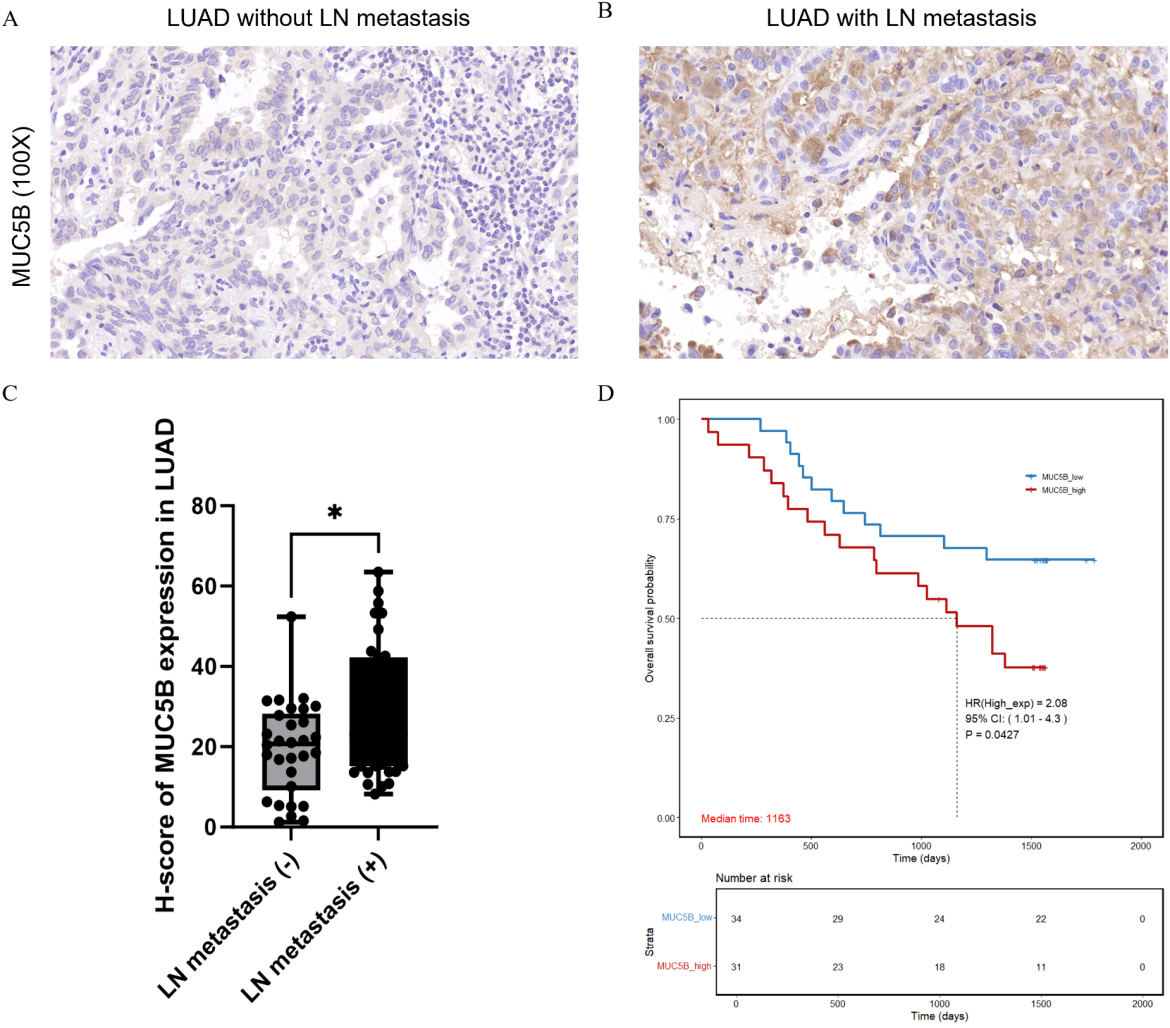
Expression and survival analysis of MUC5B in human LUAD patients. **(A, B)** Immunohistochemistry for MUC5B in LUAD without LN metastasis **(A)** and with LN metastasis **(B)**. **(C)** Quantitative analysis of H-score of MUC5B expression in LN metastasis (-) and LN metastasis (+) patients (n=30 vs. 35). **(D)** Overall survival (OS) in the high and low MUC5B groups. (*P<0.05, H-Score = Σ (pi × i) = (% weak cells × 1) + (% moderate cells × 2) + (% strong cells × 3)).

We collected tumor tissues from 65 patients with lung adenocarcinoma and performed immunohistochemical (IHC) staining to assess MUC5B expression. Among these patients, 30 had no lymph node metastasis, while 35 had lymph node metastasis. The IHC results showed that MUC5B expression was significantly higher in lung adenocarcinoma tissues with lymph node metastasis compared to those without metastasis (**Figures 8A, B**). The positive signal of MUC5B was mainly localized in the cytoplasm of lung adenocarcinoma cells, showing a granular or diffuse brownish-yellow distribution. Furthermore, we calculated the H-score for each sample, and the H-score for MUC5B expression in the group without lymph node metastasis was significantly lower than in the metastasis-positive group (P = 0.0125, **Figure 8C**). Based on the H-scores, patients were divided into two groups: MUC5B_high (top 50%) and MUC5B_low (bottom 50%). Kaplan-Meier survival analysis demonstrated that patients with higher MUC5B expression had a significantly poorer prognosis compared to those with lower expression (HR: 2.08, 95% CI: 1.01-4.3, P = 0.0427, **Figure 8D**). To avoid the impact of basic factors on survival analysis, we collected data on patients’ gender, age, TNM stage, Grade, chemotherapy, and radiotherapy, and supplemented our analysis by performing a multivariate Cox regression analysis comparing patients with high and low H-score. The results showed that only the H-score of MUC5B was a significant prognostic factor (HR: 2.42, 95%CI: 1.11–5.25, P = 0.026). These findings suggest that MUC5B may play an important role in promoting lymph node metastasis and is associated with worse overall survival in lung adenocarcinoma patients.

### 3.8 MUC5B depletion significantly inhibited cell proliferation, colony formation, migration, and invasion in lung adenocarcinoma cells

**Figure 9:**
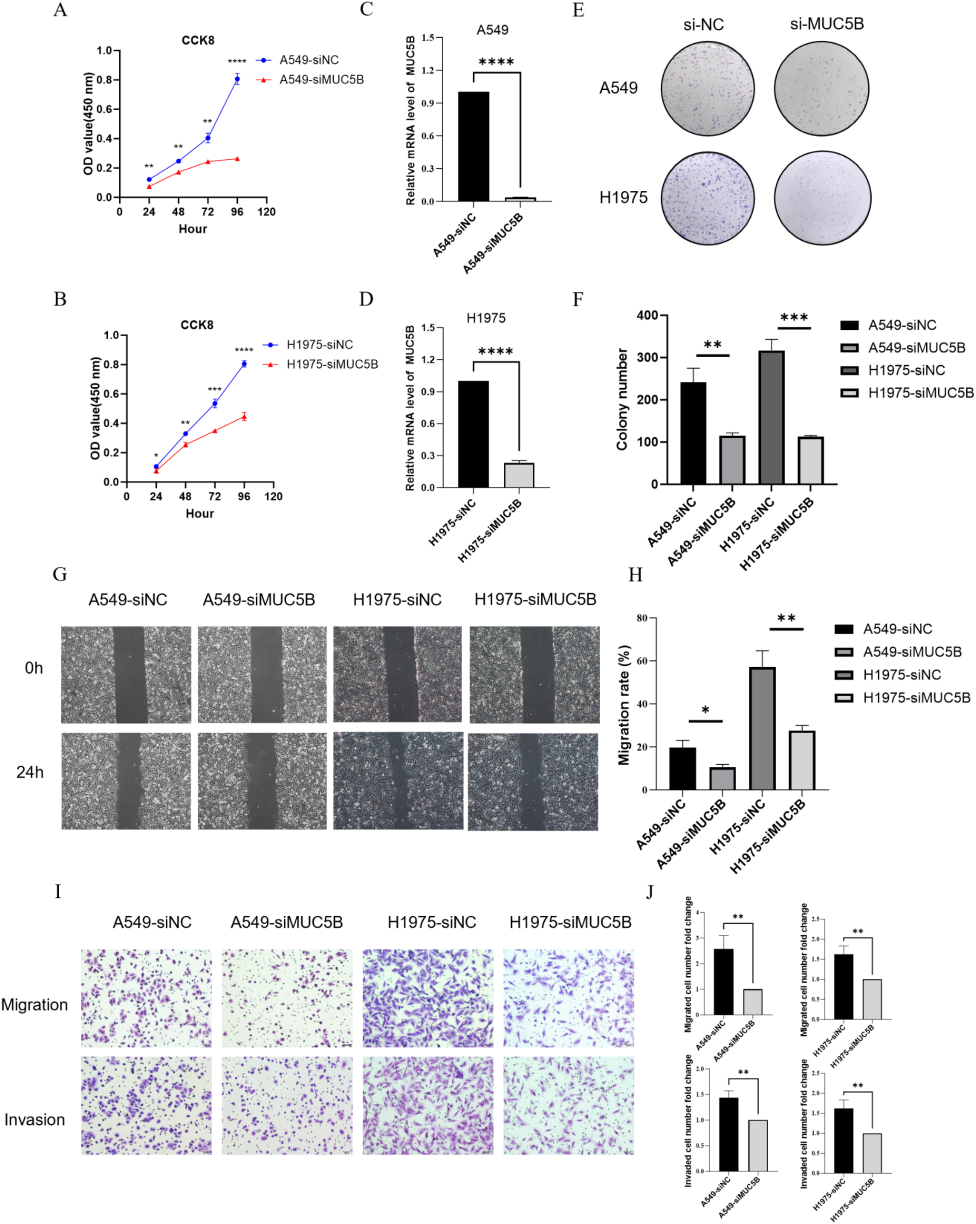
MUC5B knockdown reduces proliferation, migration, invasion, and colony formation in LUAD cells. **(A, B)** CCK-8 assays demonstrating significantly reduced cell viability in A549 and H1975 cells transfected with siMUC5B compared to siNC controls over a 96-hour period. **(C, D)** qRT-PCR results confirming the effective knockdown of MUC5B mRNA expression in A549 and H1975 cells following siMUC5B transfection. **(E)** Representative images from the colony formation assay showing significantly fewer colonies in A549 and H1975 cells transfected with siMUC5B compared to siNC. **(F)** Quantification of colony formation results, revealing a marked reduction in colony numbers in MUC5B-knockdown cells. **(G)** Wound healing assay showing impaired migration in both A549 and H1975 cells 24 hours after MUC5B knockdown. **(H)** Quantification of the wound closure rate demonstrates a significant reduction in the migration capacity of siMUC5B-transfected cells compared to controls. **(I)** Transwell migration and invasion assays showing that MUC5B knockdown significantly reduces the migratory and invasive capabilities of A549 and H1975 cells. **(J)** Quantification of migration and invasion reveals a substantial decrease in cell movement and invasiveness in MUC5B-depleted cells. Data are presented as mean ± SD. *P < 0.05, **P < 0.01, ***P < 0.001, ****P < 0.0001.

We used siRNA targeting MUC5B (siMUC5B) and a negative control siRNA (siNC) to transfect both A549 and H1975 lung adenocarcinoma cells. Cells were co-transfected with FAM-labeled siNC, and the transfection efficiency was observed to be over 80% via fluorescence microscopy at 48 hours post-transfection. qRT-PCR analysis confirmed a substantial reduction in MUC5B mRNA expression in both cell lines following siMUC5B transfection, compared to the control groups (**Figures 9C, D**). The CCK-8 viability assay demonstrated a significant decrease in the proliferation rate of both A549 and H1975 cells after MUC5B knockdown (**Figures 9A, B**). The colony formation assay revealed that MUC5B knockdown drastically inhibited the colony-forming ability of both cell lines, highlighting its key role in promoting LUAD cell proliferation (**Figures 9E, F**). In the wound healing assay, MUC5B knockdown markedly reduced the migration capacity of both.

cell lines, evidenced by decreased wound closure at 24 hours post-scratch (**Figures 9G, H**). Additionally, transwell migration and invasion assays further confirmed that MUC5B depletion led to a significant reduction in both the migratory and invasive abilities of A549 and H1975 cells (**Figures 9I, J**). Overall, these findings collectively suggest that MUC5B plays a pivotal role in facilitating lung adenocarcinoma cell proliferation, migration, invasion, and colony formation, underscoring its potential as a therapeutic target in LUAD.

### 3.9 MUC5B promotes lung adenocarcinoma progression via regulation of GINS family genes involved in DNA replication and cell cycle

**Figure 10:**
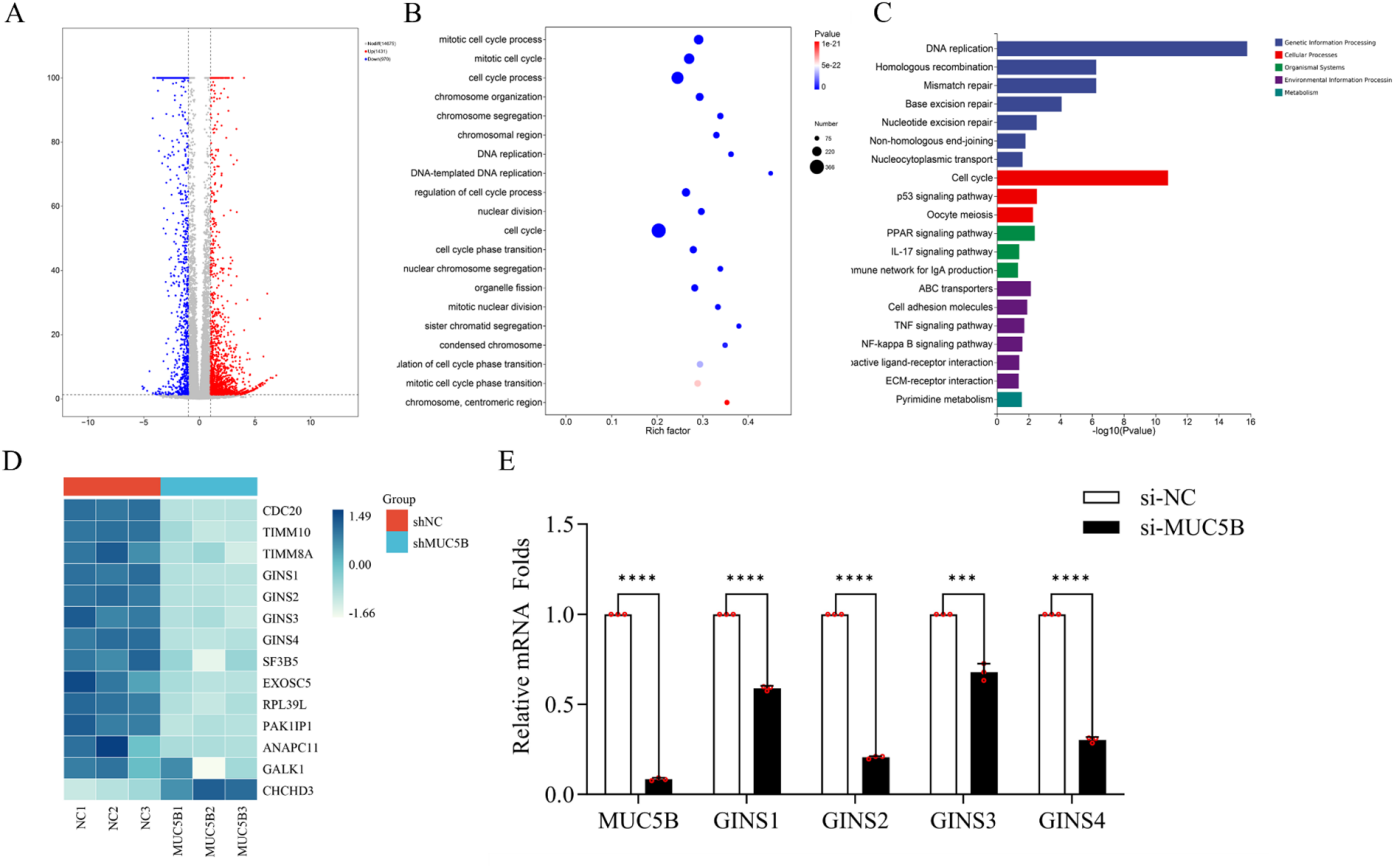
RNA-seq reveals transcriptomic changes following MUC5B knockdown in LUAD cells. **(A)** Volcano plot showing DEGs between si-MUC5B and si-NC groups. Red and blue dots represent significantly upregulated and downregulated genes (|log2FC| > 1, p < 0.05). **(B)** GO enrichment analysis of DEGs. **(C)** KEGG pathway analysis highlighting key pathways related to DNA replication and cell cycle. **(D)** Heatmap of the 13-gene model and GINS family expression after MUC5B knockdown. **(E)** qPCR validation showing reduced expression of GINS1, GINS2, GINS3 and GINS4 following MUC5B silencing in A549 cells. ***P < 0.001, ****P < 0.0001.

To explore the downstream molecular effects of MUC5B, we performed RNA-seq analysis comparing lung adenocarcinoma cells with and without MUC5B knockout. A total of 1,431 genes were upregulated and 970 genes were downregulated following MUC5B deletion (**Figure 10A**). Gene Ontology (GO) and KEGG enrichment analyses of differentially expressed genes showed significant enrichment in pathways related to DNA replication and cell cycle regulation (**Figures 10B, C**). Notably, three genes from our 13-gene predictive model—GINS1, GINS2, and GINS4—were significantly downregulated upon MUC5B knockout (**Figure 10D**). These genes are all core members of the GINS family and together with GINS3, form a heterotetrameric complex essential for DNA replication initiation and elongation (29). This observation prompted further investigation. We subsequently validated the expression of GINS1, GINS2, GINS3 and GINS4 using quantitative PCR (qPCR) in A549 cells. Consistently, all four genes exhibited markedly decreased expression in MUC5B-deficient cells. We hypothesize that MUC5B may promote lymph node metastasis in lung adenocarcinoma by modulating the expression of the GINS gene family, thereby enhancing DNA replication and cell cycle progression.

## 4 Discussion

Lung adenocarcinoma (LUAD) remains a leading cause of cancer-related mortality, with lymph node metastasis being a pivotal factor affecting prognosis and therapeutic decision-making. Therefore, elucidating the molecular mechanisms underlying LUAD metastasis and identifying robust biomarkers are essential for improving early diagnosis and advancing precision medicine. In this study, we identified MUC5B as a key driver of LUAD progression, particularly in promoting lymph node metastasis, and demonstrated its potential as both a diagnostic biomarker and therapeutic target.

Using a multi-algorithm bioinformatics pipeline combining LASSO, SVM-RFE, and WGCNA, we identified MUC5B as a central gene highly correlated with LUAD samples harboring lymph node metastasis. GO and KEGG enrichment analyses showed that MUC5B-associated genes were significantly enriched in pathways related to cell proliferation, DNA replication, cell cycle regulation, and migration. ROC curve analysis further validated the diagnostic value of MUC5B, with high expression levels correlating with advanced disease and poor prognosis. Our integrative strategy improved the specificity of metastatic gene prediction, addressing a major gap in LUAD prognosis compared to previous studies relying on single-method approaches (30–32).

MUC5B, a secreted mucin glycoprotein, has been implicated in the progression of various malignancies. In breast, colorectal, and pancreatic cancers, MUC5B overexpression is linked to tumor proliferation, invasion, and metastasis through activation of oncogenic signaling cascades such as PI3K/AKT, Wnt/β-catenin, and ERK1/2 (33–35). Additionally, in ovarian cancer, MUC5B contributes to chemoresistance via modulation of the NF-κB pathway (36). Clinical data analysis from the UCSC Xena database revealed KRAS mutation-specific variations in MUC5B expression (**Supplementary Figure 4**). Extensive research has been conducted on the role of MUC family genes in lung cancer, particularly MUC1 and MUC21. These two genes are known to affect the proliferation, immune evasion, and drug resistance of lung adenocarcinoma by disrupting cell adhesion and regulating signaling pathways such as PI3K/AKT and WNT/β-catenin (37–39). However, there is a paucity of studies focusing on the role of MUC family genes in lymph node metastasis of lung adenocarcinoma—especially regarding their function as key regulatory genes or critical biomarkers in the metastatic process. There have been several previous studies on biomarkers for lymph node metastasis in lung adenocarcinoma, with reported candidates including flotillin-1 (40), stathmin (41), and apolipoprotein E (42). However, compared with these prior works, our study not only identified MUC5B as a critical gene driving LNM in lung adenocarcinoma but also constructed a MUC5B-centered predictive model for LNM. Functional experiments revealed that MUC5B knockdown significantly suppressed LUAD cell proliferation, migration, and invasion, highlighting its oncogenic role.

Moreover, GO/KEGG enrichment results suggest that MUC5B may promote LUAD progression through three interrelated mechanisms: (1) metabolic reprogramming via mitochondrial dysfunction and galactose metabolism; (2) genomic instability through disrupted DNA replication and cell cycle control; and (3) tumor microenvironment remodeling through altered protein-DNA interactions and ubiquitin-mediated proteolysis. These mechanisms collectively support MUC5B’s capacity to enhance tumor cell survival and metastatic potential.

We constructed a 13-gene prediction model for lymph node metastasis with excellent performance (AUC > 0.9), validated in independent datasets. Notably, CDC20 and TIMM8A emerged as top contributors to model performance. CDC20 is a regulatory protein involved in the anaphase-promoting complex/cyclosome (APC/C), which plays a critical role in cell cycle progression and mitosis. Its overexpression has been linked to tumorigenesis in various cancers, including LUAD, by promoting unchecked cell division and proliferation (43, 44). The TIMM8A gene encodes a mitochondrial transport protein that is primarily involved in the translocation of proteins across the mitochondrial inner membrane, playing a crucial role in cellular energy metabolism and apoptosis (45). Interestingly, previous research has not established a direct link between TIMM8A and the pathogenesis of LUAD. TIMM8A is involved in tumor progression by regulating mitochondrial function and cellular metabolic reprogramming, exerting its effects through modulating reactive oxygen species (ROS) levels and mitochondrial respiration. This pathway may have indirect synergy with MUC5B’s function. Our study is the first to demonstrate the significant role of TIMM8A in lymph node metastasis in LUAD, providing novel insights into its potential as a therapeutic target. These findings may pave the way for future strategies aimed at preventing or treating lymph node metastasis in LUAD patients.

A striking finding from our transcriptomic analysis was the consistent downregulation of GINS1, GINS2, and GINS4 following MUC5B knockdown. These genes encode subunits of the GINS complex, a heterotetrameric DNA replication factor essential for both replication initiation and elongation (29). GINS1 promotes LUAD proliferation via Notch1/3-mediated activation of the PI3K/AKT/mTORC1 axis (46); GINS2 modulates the p53/GADD45A, STAT, and MEK/ERK pathways to enhance proliferation, migration, and epithelial-mesenchymal transition (47, 48); GINS4 has been reported to inhibit ferroptosis by suppressing p53 acetylation at K351, thereby promoting LUAD cell survival under oxidative stress (49). Our qPCR validation confirmed that MUC5B loss led to significant reductions in the expression of all three GINS genes. We hypothesize that MUC5B may exert its pro-tumor effects in LUAD partly through upregulation of the GINS complex, linking it to genomic instability and ferroptosis resistance. However, this requires further in-depth experimental mechanistic validation in the future. This newly identified MUC5B–GINS regulatory axis offers a novel mechanistic insight into LUAD progression and lymphatic metastasis.

Additionally, single-cell RNA-seq analysis revealed that MUC5B is predominantly expressed in LUAD tumor cells, particularly in those with lymph node involvement, reinforcing its specificity as a therapeutic target. In contrast, other mucins such as MUC1 and MUC21 have been reported in LUAD and NSCLC, with roles in immune modulation and EGFR mutation-specific expression patterns (50, 51), but their associations with lymph node metastasis remain less clearly defined. Our findings suggest that MUC5B may offer a more precise and metastasis-related biomarker among the mucin family.

From a translational perspective, mucin-targeted therapies have shown clinical promise. For example, MUC16 (CA125) is widely used in ovarian cancer diagnostics (52). Monoclonal antibodies such as Oregovomab and BiTE molecules like REGN4018, targeting MUC16 and CD3, have demonstrated encouraging results in early-phase trials (53, 54). Given MUC5B’s tumor-specific expression and strong link to metastatic potential, targeted inhibition of MUC5B may offer a novel strategy for LUAD patients, especially those with advanced-stage disease. In this study we constructed a lymph node metastasis prediction model based on lung adenocarcinoma tumor samples; for clinical patients undergoing needle biopsy for small biopsy samples—for whom mediastinal or hilar lymph node biopsy is not feasible—this model can effectively assess the risk of lymph node metastasis.

However, this study has limitations. Most data are derived from *in vitro* analyses and retrospective datasets; thus, *in vivo* validation using LUAD animal models is needed. The scRNA-seq and validation datasets lack lymph node tissue data. It will be necessary to further validate our research findings in lymph node samples and conduct in-depth exploration of the mechanisms underlying lymph node metastasis in the future. Moreover, although we identified several candidate genes downstream of MUC5B, the exact transcriptional regulation mechanisms require further exploration. Large-scale prospective clinical cohorts and functional experimental verification at the animal level will be essential to validate the prognostic value of MUC5B and assess the efficacy of potential MUC5B-targeted therapies in the future.

## 5 Conclusions

In conclusion, this study provides strong evidence that MUC5B plays a critical role in promoting lymph node metastasis and poor prognosis in LUAD. MUC5B overexpression in metastatic LUAD tissues, along with its ability to enhance cell proliferation, migration, invasion, and colony formation, underscores its potential as both a biomarker and therapeutic target. Targeting MUC5B could offer new opportunities for improving the diagnosis, treatment, and prognosis of LUAD patients, particularly those with advanced metastatic disease. By promoting GINS complex expression and driving cell cycle progression and ferroptosis resistance, MUC5B contributes to aggressive tumor behavior. These insights into the MUC5B–GINS axis and associated oncogenic pathways provide a foundation for future studies aiming to improve diagnostic accuracy and develop targeted therapies for LUAD.

## Data Availability

The data presented in the study are deposited in the NCBI Sequence Read Archive (SRA) repository, accession number PRJNA1358950 (https://www.ncbi.nlm.nih.gov/bioproject/PRJNA1358950/).
