# Supplementary material for: Identification of MUC5B as a lymph node metastasis-associated gene in lung adenocarcinoma through integrated transcriptomic and machine learning approaches
Source: Front Immunol. 2025 Dec 2;16:1666240. doi: 10.3389/fimmu.2025.1666240 (PMC12705602; doi:10.3389/fimmu.2025.1666240)
Supplement: Supplementary file 1 [file Table1.docx]

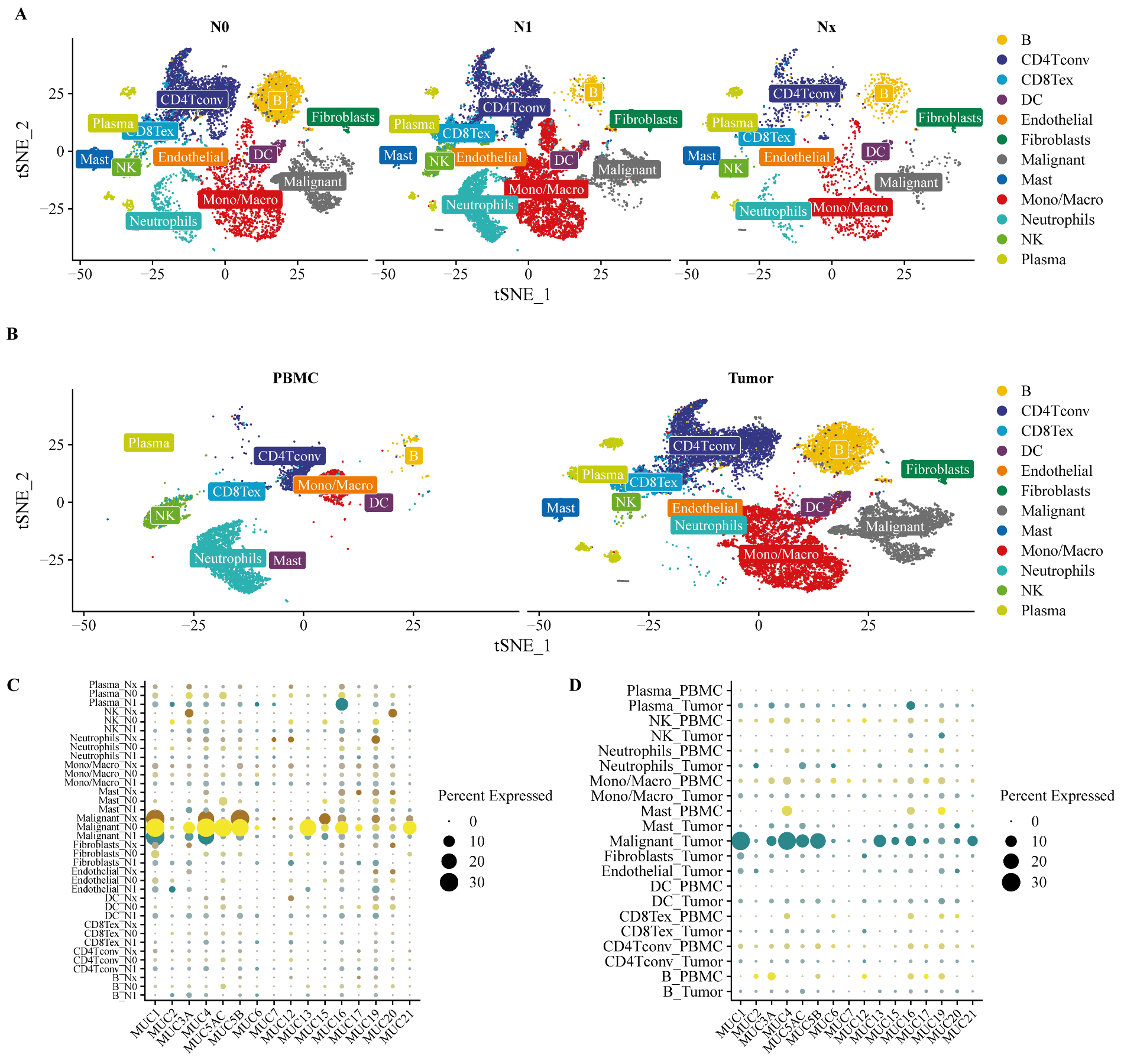
Supplementary Fig. 1 Single-cell transcriptomic analysis of tumor and immune cells in different metastatic statuses. (A) t-SNE plots of cell populations in N0, N1, and Nx groups; (B) t-SNE plots comparing peripheral blood mononuclear cells (PBMC) and tumor-infiltrating cells; (C) Dot plot showing MUC family gene expression across cell types in N0, N1, and Nx groups; (D) Dot plot comparing MUC family gene expression between PBMCs and tumor-infiltrating cells.


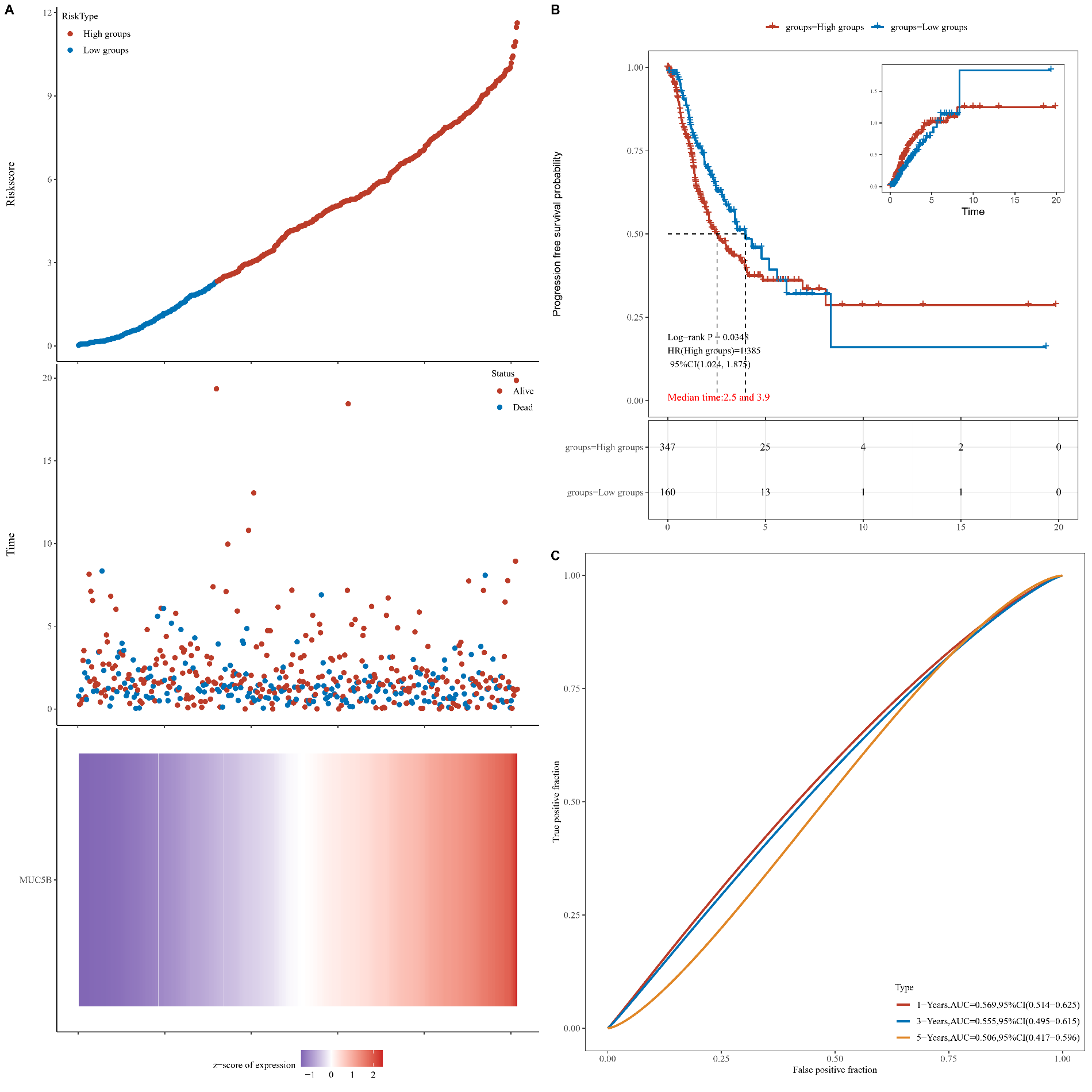
Supplementary Fig. 2 Survival analysis and predictive performance evaluation based on risk stratification. (A) Risk score distribution, survival status, and MUC5B expression; (B) Survival curve analysis; (C) ROC curve for predictive model.


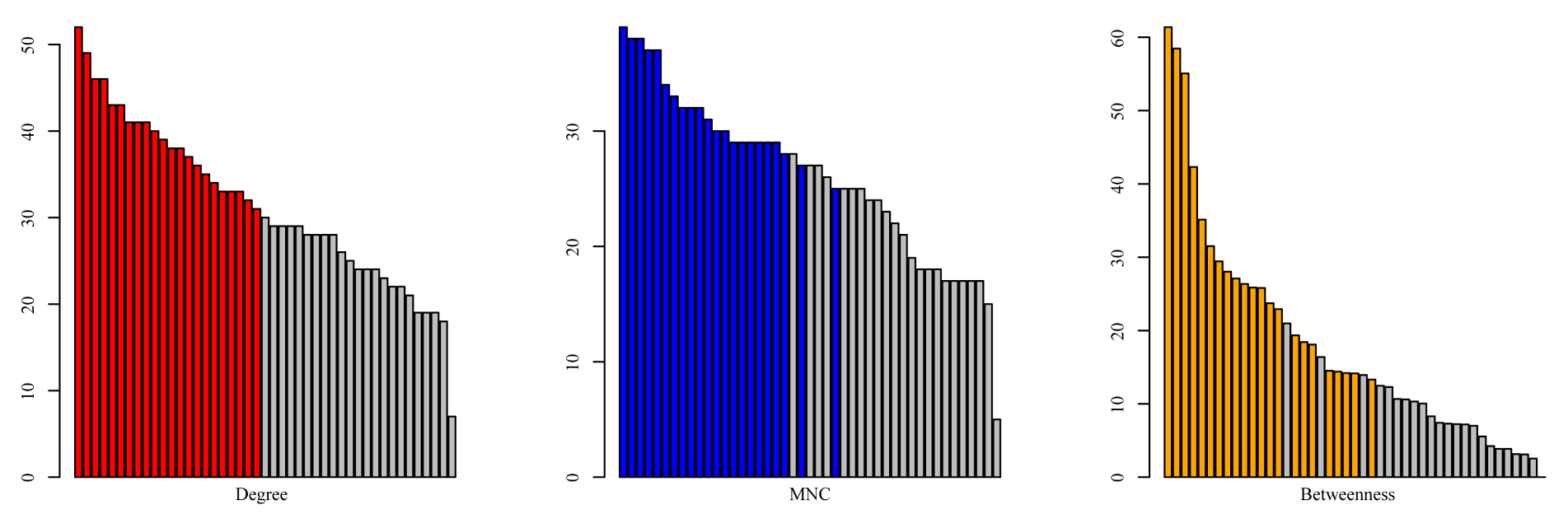
Supplementary Fig. 3 Node importance ranking based on different network centrality measures (Degree, MNC and Betweenness).


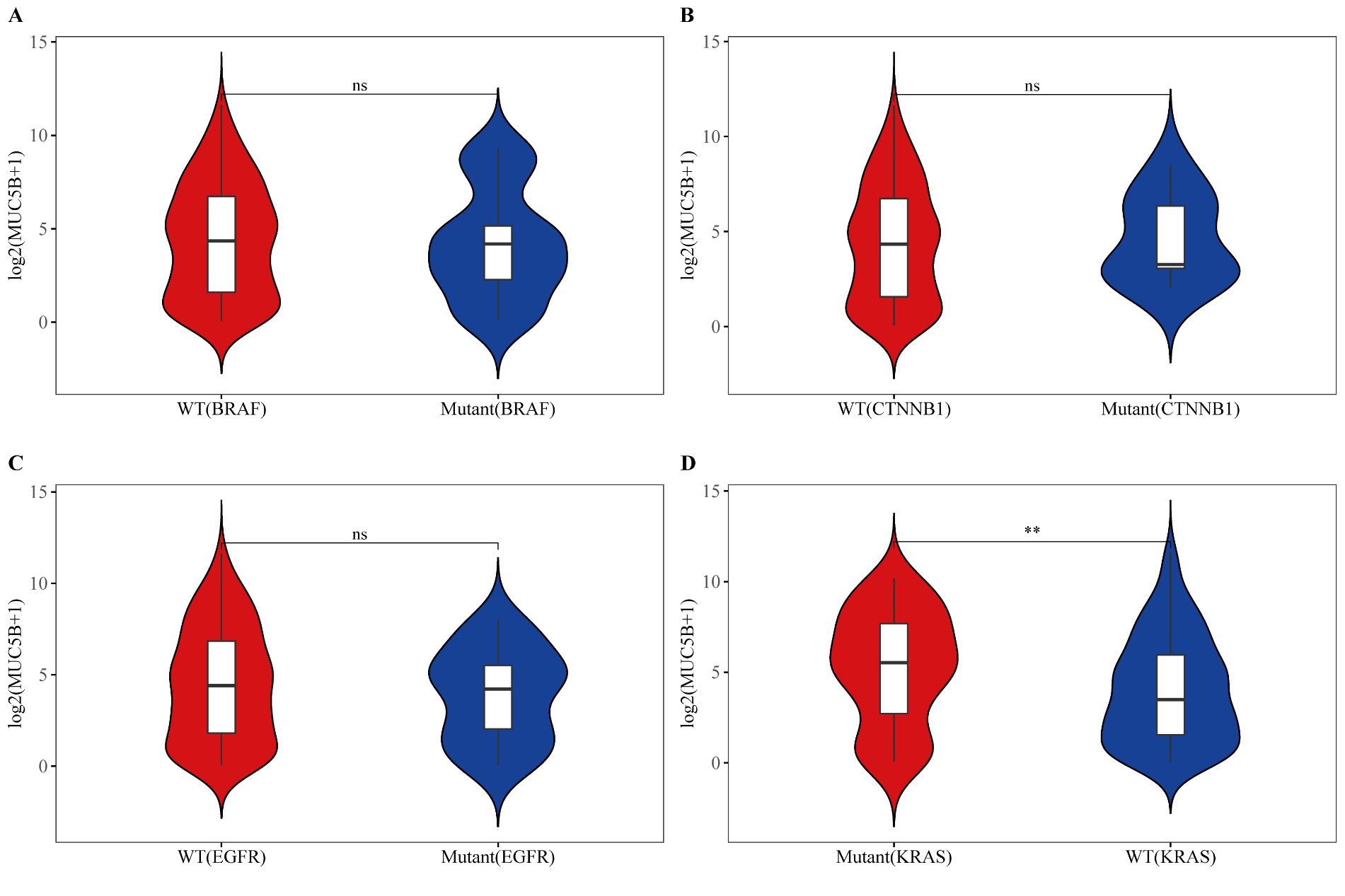
Supplementary Fig. 4 MUC5B expression in different gene mutation status. (A) Comparison of MUC5B expression between BRAF wild-type (WT) and mutant groups; (B) Comparison of MUC5B expression between CTNNB1 wild-type and mutant groups; (C) Comparison of MUC5B expression between EGFR wild-type and mutant groups; (D) Comparison of MUC5B expression between KRAS wild-type and mutant groups. (ns: not significant; **: *P*<0.01)
